# Temporal changes in the neutrophil to lymphocyte ratio and the neurological progression in cryptogenic stroke with active cancer

**DOI:** 10.1371/journal.pone.0194286

**Published:** 2018-03-16

**Authors:** Ki-Woong Nam, Tae Jung Kim, Chi Kyung Kim, Heejung Mo, Han-Yeong Jeong, Min Kyoung Kang, Moon-Ku Han, Sang-Bae Ko, Byung-Woo Yoon

**Affiliations:** 1 Department of Neurology, Seoul National University College of Medicine, Seoul, Korea; 2 Department of Neurology, Seoul National University Hospital, Seoul, Korea; 3 Department of Neurology, Korea University Guro Hospital and Korea University College of Medicine, Seoul, Korea; 4 Department of Neurology, Seoul National University Bundang Hospital, Seongnam, Korea; Massachusetts General Hospital, UNITED STATES

## Abstract

**Background:**

Ischemic stroke patients with active cancer frequently experience early neurological deterioration (END); however, the predictors of END are not well studied. The neutrophil to lymphocyte ratio (NLR) has recently been described as a predictor of poor outcomes in cancer and stroke. However, its role in cancer-related stroke has not been addressed.

**Aim:**

We aimed to evaluate the association between the NLR and END in cancer-related stroke patients.

**Methods:**

We included 85 cryptogenic stroke patients with active cancer. END was defined as an increase ≥ 4 on the total National Institutes of Health Stroke Scale (NIHSS) score within 72 hours of admission. The NLR was calculated as the ratio of the absolute neutrophil count to the absolute lymphocyte count. We obtained the NLR during the following three periods: at admission, 1–3 days after admission (D 1–3 NLR) and 4–7 days after admission (D 4–7 NLR).

**Results:**

END occurred in 15 (18%) of the 85 patients. END was significantly associated with the initial NIHSS score, infarction volume, and the D 1–3 NLR. In multivariate analysis, a higher D 1–3 NLR, measured before END events, remained an independent predictor of END [adjusted odds ratio = 2.78, 95% confidence interval = 1.09–7.08, *P* = 0.032]. In terms of temporal changes in the NLR, the END group showed a tendency toward temporal increase in the NLR at D 1–3 (*P* = 0.061) with subsequent decrements in the D 4–7 NLR (*P* = 0.088), while the non-END group showed no significant changes in the NLR between periods.

**Conclusions:**

This study demonstrated that a higher NLR could predict END events in cryptogenic stroke patients with active cancer. However, the results should be confirmed in further large prospective studies.

## Introduction

Ischemic stroke is common among cancer patients and indicates a poorer prognosis for such patients [1,2]. The mechanisms of stroke in this group are more complex, especially when both conventional vascular risk factors and cancer-specific factors related to thrombogenicity are involved [3,4]. Due to these heterogeneous traits, cancer-related stroke may result from different stroke mechanisms and require specific treatments. However, despite their clinical importance, studies on cancer-related stroke and its early clinical outcomes have not been well addressed.

Early neurological deterioration (END) following ischemic stroke is a clinically important event because it is strongly related to a subsequent poor prognosis. Various clinical and systemic factors have been suggested as predictors of END, including metabolic and hemodynamic factors, initial stroke severity, and inflammation. Inflammatory markers could also be predictors of END in cancer-related stroke, since the inflammatory process is important in both cancer and stroke [5–8]. Inflammation is involved in carcinogenesis and in cancer progression and metastasis, all of which affect the microenvironment of cancer cells [9,10]. Additionally, inflammation determines the infarct size and early neurological outcomes, thereby threatening the ischemic penumbra in stroke patients [6,11,12].

The neutrophil to lymphocyte ratio (NLR) is a marker of systemic inflammation, and it has proven to be a poor prognostic marker in cancer patients [5,13,14]. Activated neutrophils secrete various tumor growth-promoting factors [10,15], and relative lymphopenia indicates attenuated host cell-mediated immunity to cancer cells [10,16]. A high NLR is commonly found in advanced cancer patients [17,18], and activated neutrophils also enhance thrombogenesis or platelet aggregations [19]. Furthermore, the NLR has recently emerged as a prognostic marker in cardiovascular disease and stroke [20–22]. Therefore, the NLR may be associated with END in cancer-related stroke patients, but this has not been studied.

In this study, we aimed to assess the association between the NLR and END in cryptogenic stroke patients with active cancer. Furthermore, we evaluated the effects of temporal variations in the NLR to suggest clues regarding the inflammatory pathophysiology of END.

## Materials and methods

### Patients and population

As part of a consecutive registry in two large centers in Korea (Seoul National University Hospital and Seoul National University Bundang Hospital) between March 2011 and June 2015, we were able to collect data of ischemic stroke patients with active cancer within 72 hours of symptom onset (n = 158). Since our main outcome, END events, is a variable with binary characteristics, the pre-determined sample size and the period of review were determined using the rule of ten [23]. We presumed an event rate of approximately 30% according to a previous study [24]. Active cancer was defined as a new diagnosis, treatment, progression, or recurrence of cancer within the 6 months prior to enrollment [1,25]. Since the ischemic stroke patients with active cancer were in poor medical conditions, we conducted multiple laboratory examinations during acute periods of stroke, as determined by each patient’s physician. Among these patients, we selected those with cryptogenic stroke that was known to have a cancer-specific stroke mechanism, excluding conventional mechanisms (i.e., large artery atherosclerosis, small vessel occlusion, cardioembolism, and other determined etiology) based on the Trial of Org 10172 in Acute Stroke Treatment (TOAST) classification (n = 120).

Moreover, we excluded patients with the following conditions: lacking complete blood cell count data (n = 31); having a history of hematologic disease or primary hematologic malignancy, which have stroke mechanisms different from those in solid cancer and that directly affect the blood cell count data (n = 4) [24]. Finally, 85 patients were included in the analyses. This study was approved by the institutional review board at Seoul National University Hospital (IRB no. 1009-062-332).

### Clinical assessment

We evaluated the baseline demographic, clinical, and vascular risk factors (i.e., age, sex, hypertension, diabetes mellitus, hyperlipidemia, and current smoking) [24]. Data regarding cancer were also assessed, including cancer type, systemic metastasis, brain metastasis, presence of adenocarcinoma, and history of venous thrombosis. Treatment options were evaluated by initial anti-thrombotic agents (none, anti-coagulant, anti-platelet, and both) initial thrombolysis therapy (none, intravenous thrombolysis, intraarterial thrombectomy, and both) and cancer-related therapy (chemotherapy, radiotherapy, surgery, and no therapy).

Stroke severity was assessed daily from admission to the discharge date by certified neurologists, who were not included in the current study, using the National Institutes of Health Stroke Scale (NIHSS) score. END was defined as an increase ≥ 4 on the total NIHSS score within 72 hours of admission [24,26,27]. The timing of END was also captured based on the review of medical records. To confirm the effects of END on subsequent outcomes, we additionally evaluated the 30-day mortality, hospitalization duration, and 3-month modified Rankin Scale (mRS) scores.

Laboratory examinations were conducted within 24 hours of admission, including blood cell counts and measurement of C-reactive protein and D-dimer levels. Blood cell samples were collected in a calcium ethylene diamine tetra-acetic acid (EDTA) tube and immediately centrifugated (2,000 rpm for 20 minutes at 4°C). The cell count analyses were performed with an autoanalyzer (XE-2100, Sysmex, Kobe, Japan) in our centers. The NLR was calculated as the ratio of the absolute neutrophil count over the absolute lymphocyte count (neutrophil count/lymphocyte count) [28]. Since this study also focused on the effects of temporal variations in the NLR on END, we obtained the NLRs from all participants during the following three periods to consider the temporal relationship between the NLR values and END: at admission (initial NLR), 1–3 days after admission just before END events (D 1–3 NLR), and 4–7 days after admission (D 4–7 NLR) (i.e., after END events). Due to the retrospective nature of the study, we present the D 1–3 NLR and the D 4–7 NLR as the mean values when patients had multiple blood cell data during these periods.

The blood cell count data were easily interrupted by infection events. Thus, we additionally assessed infection events as a variable. An infection event was defined as any type of infection that was clinically diagnosed by physicians (i.e., pneumonia, urinary tract infection, septic shock, gastrointestinal infection) during the acute period.

### Radiological assessment

All participants underwent magnetic resonance imaging (MRI) and magnetic resonance angiography (MRA) conducted with a 3.0-Tesla MR scanner (Achieva 3.0T; Philips, Eindohovenm, the Netherlands) within 24 hours of admission. We performed broad MRI acquisition as follows: diffusion-weighted imaging (DWI) [repetition time (TR)/echo time (TE) = 4800/66 or 6300/80 ms], T1-weighted imaging [TR/TE = 300/10 or 500/11 ms], T2-weighted imaging [TR/TE = 4800/100 or 5000/127 ms], fluid-attenuated inversion recovery imaging [TR/ TE = 11,000/140 or 8800/127 ms], T2 gradient echo imaging [TR/TE = 28/20 or 57/20 ms], and three-dimensional time of flight MRA imaging [TR/TE = 20/7 ms, slice thickness = 1.2 mm]. The basic slice thickness was 5.0 mm in the axial plane, except in the flight MRA images. The locations of index DWI lesions were classified as anterior circulation, posterior circulation, or both. We also dichotomized the initial DWI lesion patterns as single territory lesions or multiple territory lesions, since multiple territory lesions showed worse outcomes in previous studies of cancer-related stroke [1]. We also rated the volume of the initial DWI lesions using Medical Imaging Processing, Analysis, and Visualization (MIPAV, version 7.3.0, National Institutes of Health, Bethesda, MD, USA), as conducted by an investigator blinded to the clinical information.

### Statistical analysis

We present the normally distributed data as the mean ± SD, and the other data are presented as the median + interquartile range. Continuous variables with skewed data were transformed into a log-scale. To compare the baseline characteristics between groups with and without END, univariate analyses were conducted using either Student’s *t*-test or Mann-Whitney *U*-test for continuous variables and a chi-squared test or Fisher’s exact test for categorical variables. Then, we conducted a multivariate analysis using binary logistic regression analysis, including variables with *P* < 0.05 in univariate analysis and infection events as confounders.

To assess the temporal relationship between the NLR and END, we compared the NLR values during all periods between the patient groups both with and without END. Additionally, we used a Wilcoxon Rank test for assessing meaningful changes in the NLR between admission to D 1–3 and the D 1–3 to D 4–7 periods. All statistical analyses were conducted using SPSS version 23 (IBM SPSS, Chicago, IL, USA), and variables with *P* < 0.05 were considered significant.

## Results

A total of 85 patients were enrolled (mean age of 68 years, time delay from symptom onset to visit = 5 [1.5–25] hours, initial NIHSS score = 5 [3–15]). The median NLR of the included patients was 5.28 [3.15–9.35] at admission, 7.12 [3.77–10.11] at D 1–3, and 6.55 [3.67–10.64] at D 4–7. The END events occurred in 15 (18%) patients and the mean time from admission to END events was 1 [1–1] day. The infection events occurred in 16 (19%) participants [median time from admission: 4.5 days], and 1 (6%) of them occurred before END events. The baseline characteristics between with and without END groups are presented in Table 1 and Table in S1 Table. The END group showed significantly higher initial NIHSS scores, larger DWI volumes, lower D 1–3 lymphocyte counts and higher D 1–3 neutrophil counts and D 1–3 NLRs (Table 1).

**Table 1:** Baseline characteristics of patients with and without END.

	No END (n = 70)	END (n = 15)	P value
Age, y [SD]	68 ± 11	71 ± 14	0.423
Visit time, h [IQR]	5.0 [1.5–24]	6 [1–45]	0.690
Sex, male, %	38 (54)	10 (67)	0.380
Hypertension, %	39 (56)	8 (53)	0.866
Diabetes, %	13 (19)	5 (33)	0.204
Hyperlipidemia, %	12 (17)	5 (33)	0.155
Current smoking, %	22 (31)	6 (40)	0.522
Venous thrombosis, %	8 (11)	3 (20)	0.401
Cancer type, %			0.859
Lung	14 (20)	3 (20)	
Stomach	12 (17)	2 (13)	
Gastrointestinal	7 (10)	0 (0)	
Hepatobiliary	19 (27)	6 (40)	
Genitourinary	10 (14)	2 (13)	
Prostate	4 (6)	1 (7)	
Breast	2 (3)	0 (0)	
Others	2 (3)	1 (7)	
Systemic metastasis, %	59 (84)	11 (73)	0.454
Brain metastasis, %	8 (11)	1 (7)	1.000
Adenocarcinoma, %	49 (74)	8 (62)	0.350
Cancer treatments			
Chemotherapy, %	48 (69)	9 (60)	0.522
Radiotherapy, %	13 (19)	4 (27)	0.487
Surgery, %	27 (39)	4 (27)	0.556
No treatment, %	12 (17)	5 (33)	0.155
Initial NIHSS score [IQR]	5 [2–11]	15 [12–18]	0.001
Initial treatment, %			0.593
No	1 (1)	1 (7)	
Anti-coagulant	38 (54)	9 (60)	
Anti-platelet agent	26 (37)	4 (27)	
Both	5 (7)	1 (7)	
Initial thrombolysis, %			0.173
No	62 (89)	11 (73)	
Intravenous	0 (0)	0 (0)	
Intraarterial	6 (9)	2 (13)	
Both	2 (3)	2 (13)	
Infection event, %	13 (19)	3 (20)	1.000
D-dimer, μg/mL [IQR]*	5.31 [1.80–16.86]	17.22 [4.96–20.00]	0.056
C-reactive protein, mg/dL [IQR]*	5.53 [1.21–10.30]	8.50 [2.30–13.18]	0.222
Initial NLR [SD]*	6.97 ± 6.43	12.48 ± 13.62	0.128
D 1–3 NLR [SD]*	7.54 ± 6.26	16.23 ± 12.226	0.002
D 4–7 NLR [SD]*	8.42 ± 8.36	11.94 ± 7.72	0.050
MRI lesion location, %			0.254
Anterior circulation	26 (37)	7 (47)	
Posterior circulation	11 (16)	0 (0)	
Both	33 (47)	8 (53)	
MRI lesion pattern, %			0.766
Single territory	23 (33)	4 (27)	
Multiple territory	47 (67)	11 (73)	
Initial DWI volume, mL [IQR]	11.26 [1.43–22.03]	32.61 [4.58–80.41]	0.021
Hemorrhagic transformation, %	9 (13)	4 (27)	0.232

*These variables were transformed into a log scale

END = Early neurological deterioration, NIHSS = National Institutes of Health Stroke Scale, NLR = Neutrophil to lymphocyte ratio, MRI = Magnetic resonance imaging, DWI = Diffusion-weighted imaging

In the multivariate analysis, the D 1–3 NLR measured before END events, remained an independent predictor of END [adjusted odds ratio (aOR) = 2.78, 95% confidence interval (CI) = 1.09–7.08, *P* = 0.032, Table 2]. To adjust for the interaction between the NLR and infection, we additionally introduced infection events as a confounder, and the D 1–3 remained significant (aOR = 2.91, 95% CI = 1.12–7.60, *P* = 0.029). These results also remained significant when we conducted an additional multivariate analysis that included the initial DWI volume instead of the initial NIHSS score (Table in S2 Table).

**Table 2:** Multivariate analysis of the possible predictors of END.

	Univariate analysis		Model 1^a^		Model 2^b^	
	OR	*P*	aOR	*P*	aOR	*P*
Infection	1.10 [0.27–4.45]	0.898	…	…	0.30 [0.05–1.70]	0.172
Initial NIHSS	1.13 [1.04–1.22]	0.002	1.08 [0.99–1.18]	0.082	1.11 [1.00–1.23]	0.044
D 1–3 NLR^C^	3.87 [1.65–9.09]	0.002	2.78 [1.09–7.08]	0.032	2.91 [1.12–7.60]	0.029

NIHSS = National Institutes of Health Stroke Scale, NLR = Neutrophil to lymphocyte ratio

^a^Adjusted for the initial NIHSS score and D 1–3 NLR

^b^Adjusted for the initial NIHSS score, D 1–3 NLR, and infection event

^C^This variable was transformed into a log scale

**Fig 1:**
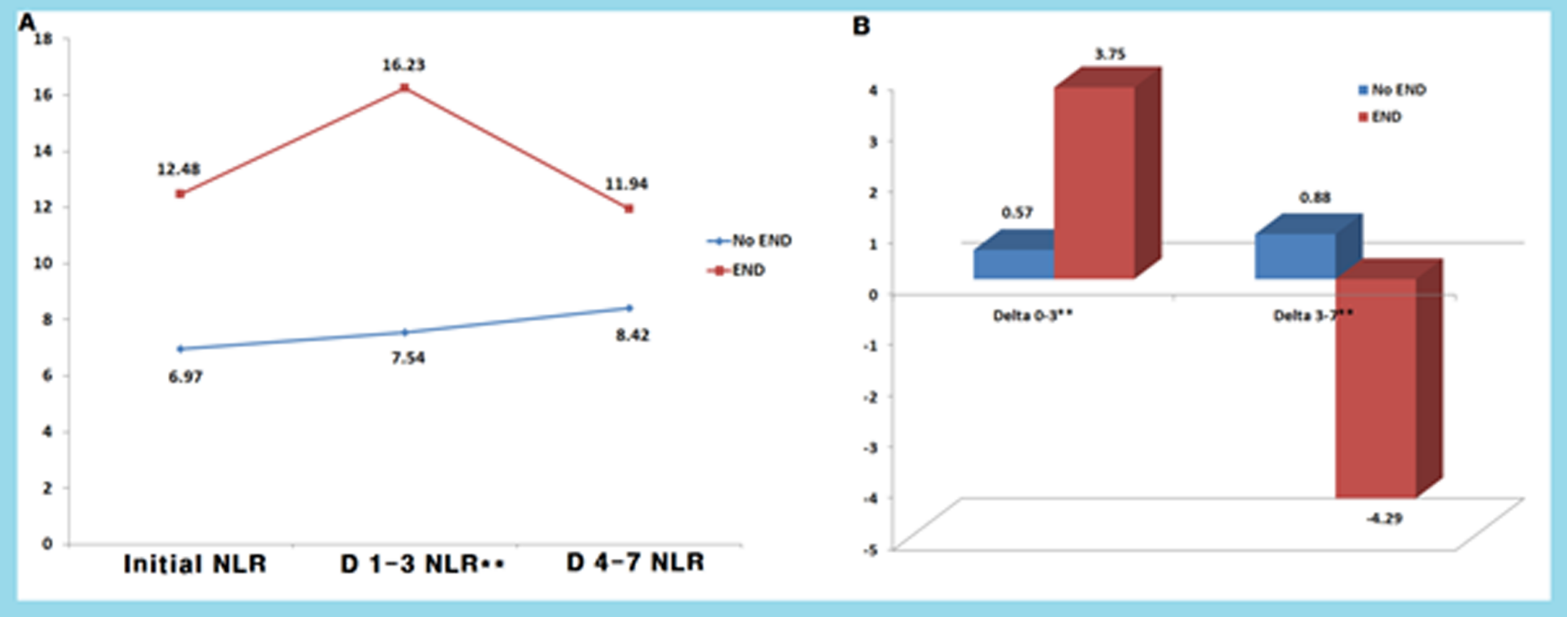
Dynamic changes in the NLR between the with and without END groups. The END group showed consistently higher NLRs than the non-END group, especially in the D 1–3 NLR (Mann-Whitney test, *P* = 0.002), with a tendency toward a transient increase at D 1–3 (A). Differences in the NLR values between admission and the D 1–3 NLR and between the D 1–3 NLR and the D 4–7 NLR were also significant between the with and without END groups (*P* = 0.036 and *P* = 0.025, respectively) (B).

In terms of temporal changes in the NLR, the END group showed consistently higher NLR values than the non-END group, especially in the D 1–3 NLR, which was obtained before the development of the END events (*P* = 0.002) (Figure in S1 Fig). Additionally, the END group showed a tendency toward a temporal increase in the NLR at D 1–3 (*P* = 0.061), with subsequent decrements over time until the D 4–7 NLR (*P* = 0.088) based on Wilcoxon Rank test. Meanwhile, the non-END group showed no significant changes in the NLR between admission and the D 1–3 NLR (*P* = 0.126) and between the D 1–3 and the D 4–7 NLR (*P* = 0.307) (Fig 1). Thus, the differences in the NLR values between admission and the D 1–3 NLR and between the D 1–3 NLR and the D 4–7 NLR were significantly different between the groups with and without END (0.57 versus 3.75, *P* = 0.036: 0.88 versus -4.29, *P* = 0.025, respectively). These tendencies were not present when they were accompanied by infection events (Figure in S2 Fig).

Subsequent outcomes after the END events are compared in Table 3. The END group showed significantly higher 3-month mRS scores than the non-END group (*P* = 0.046). There was a tendency of higher 30-day mortality rates and longer hospitalization durations in the END group that did not reach statistical significance (*P* = 0.079 and 0.077, respectively). These clinical outcomes were not different among different cancer types (*P* = 0.867 in 30-day mortality; *P* = 0.906 in 3-month mRS; *P* = 0.279 in hospitalization duration).

**Table 3:** Clinical outcomes between the with and without END groups.

	No END (n = 70)	END (n = 15)	P value
3-Month mRS score	6 [2–6]	6 [6–6]	0.046
30-Day mortality, %	10 (14)	5 (33)	0.079
Hospitalization duration, d [IQR]	13 [9–20]	23 [13–29]	0.077

mRS = modified Rankin Scale

## Discussion

In this study, we found that a high D 1–3 NLR, which was obtained just before an END event, was independently associated with END in cryptogenic stroke patients with active cancer. This finding suggests that inflammation may be one of the pathologic mechanisms in END events because the NLR is a marker of systemic inflammation.

We have several possible explanations for the relationship between a high NLR and END events. First, one explanation is related to the severity of the index stroke. We already know that severe stroke and large initial DWI volumes are potent predictors of END [29–31]. When large and severe strokes occur, the activated neutrophils infiltrate the ischemic areas and enlarge damaged tissues, releasing various destructive materials (i.e., proteolytic enzymes, oxygen free radicals, arachidonic acids, and elastase) during the acute phase [20,21]. Meanwhile, lymphocyte counts decrease in response to corticosteroids secondary to stressful stroke events [19]. As a result, severe strokes have larger inflammation burdens and could elevate the NLR [32]. Consistent with previous studies, we found close correlations between the initial NIHSS score or initial DWI volume and the NLR during all the periods (initial, D 1–3, and D 4–7) in the cohort. Second, adverse events after the index stroke may lead to neurological progression and aggravate additional inflammatory responses. END results from various mechanisms (i.e., stroke recurrence, progression of index lesion, edematous change, hemorrhagic transformation) [33], and these adverse events accompany subsequent inflammation. Since there is a temporal difference in the activation timing between neutrophils [6 to 24 hours] and lymphocytes [7 days] during the inflammatory process [21], the NLR may be elevated shortly after the adverse event and slowly decrease over time. In support of this idea, we found that the D 1–3 NLR was mostly correlated with END events and that a transient increasing tendency in the NLR values occurred just before END. Finally, a higher NLR suggests a hostile environment, which is vulnerable to ischemic insult. The NLR might be a marker of subclinical inflammation that is related to chronic diseases, including various metabolic diseases, cardiovascular disease, cerebral small vessel diseases and atherosclerosis [28,34–36]. Patients with a higher NLR may be more vulnerable to ischemic insults and could have frequent END events.

To interpret our results, infection factors should also be considered. Stroke and cancer patients had frequent infection events during hospitalization [37,38], which could directly affect the NLR values. Furthermore, non-neurological medical complication after stroke is one of the mechanisms of neurological progression. Thus, one may think that the close relationship between a high NLR and END simply reflects concurrent infection events. However, most of the infection events (94%) occurred after END events, and the NLR remained significant after adjusting for infection events. Additionally, the increase in the NLR just before END events (D 1–3 NLR) disappeared when it was accompanied by an infection. Thus, we thought there may be pathophysiologic mechanisms related to the connection between the NLR and END other than infection.

This study has several caveats. First, this study was designed as a retrospective study based on two centers, so selection bias is possible, and the results should be generalized with caution. However, the consecutive series of cryptogenic stroke patients with active cancer in our study had full NLR data during all three periods, and these data were relatively homogenous. Thus, we think the present study has enough significance to guide further studies. Second, we included patients who visited within 72 hours of symptom onset. Thus, there is a possibility that underevaluation of END occurred during the preadmission period. However, the median time delay to visit was 5 hours, and 75% of the participants visited within 24 hours, and therefore, the proportion of patients in whom END was missed may not be considerable. Last, we included miscellaneous solid cancer patients using relatively simple cancer staging methods. The effects of the type or stage of cancer may be underestimated, in contrast with the close relationship between advanced cancer and the NLR established in previous studies [9,17]. Thus, the results should be generalized to clinical fields with caution, and further studies on single tumors that use detailed staging are warranted.

In conclusion, we found that a high NLR may predict END events in cryptogenic stroke patients with active cancer, especially when they measured just before the events. Because the NLR is easily obtainable from a blood test, it might be helpful in identifying high-risk patients that merit further examination. However, the results of the present study should be confirmed in further large prospective studies.

## Supporting information

S1 Table Temporal changes in blood cell count data between groups with and without END. (DOCX)

S2 Table Multivariate analysis of the possible predictors of END. (DOCX)

S1 Fig NLR values between with and without END group during three periods. (TIF)

S2 Fig Dynamic changes in the NLR between the with and without END groups and an infection event. The END group showed a consistently higher NLR than the non-END group, especially the D 1–3 NLR (Mann-Whitney test, *P* = 0.001), with a tendency toward increase at D 1–3 (A, B). However, these differences were affected by infection events during hospitalization (C, D).
(TIF)
