# Supplementary material for: Temporal changes in the neutrophil to lymphocyte ratio and the neurological progression in cryptogenic stroke with active cancer
Source: PLoS One. 2018 Mar 16;13(3):e0194286. doi: 10.1371/journal.pone.0194286 (PMC5856344; doi:10.1371/journal.pone.0194286)
Supplement: S1 Table — (DOCX) [file pone.0194286.s001.docx]

S1 Table Temporal changes in blood cell counts data between groups with and without END

|  | **No END (n = 70)** | **END (n = 15)** | **P value** |
| --- | --- | --- | --- |
| NLR |  |  |  |
| Initial NLR [SD]* | 6.97 ± 6.43 | 12.48 ± 13.62 | 0.128 |
| D 1-3 NLR [SD]* | 7.54 ± 6.26 | 16.23 ± 12.226 | 0.002 |
| D 4-7 NLR [SD]* | 8.42 ± 8.36 | 11.94 ± 7.72 | 0.050 |
|  |  |  |  |
| White blood cell counts |  |  |  |
| Initial WBC [SD] | 8.88 ± 4.99 | 10.83 ± 5.05 | 0.114 |
| D 1-3 WBC [SD] | 8.63 ± 5.22 | 10.45 ± 4.32 | 0.084 |
| D 4-7 WBC [SD] | 9.86 ± 6.81 | 11.55 ± 6.13 | 0.143 |
|  |  |  |  |
| Neutrophil counts |  |  |  |
| Initial neutrophil [SD] | 6.83 ± 4.33 | 8.73 ± 5.29 | 0.167 |
| D 1-3 neutrophil [SD] | 6.69 ± 4.61 | 8.73 ± 3.99 | 0.033 |
| D 4-7 neutrophil [SD] | 7.36 ± 5.22 | 9.45 ± 5.55 | 0.109 |
|  |  |  |  |
| Lymphocyte counts |  |  |  |
| Initial lymphocyte [SD] | 1.21 ± 0.62 | 1.16 ± 0.78 | 0.426 |
| D 1-3 lymphocyte [SD] | 1.07 ± 0.51 | 0.79 ± 0.47 | 0.040 |
| D 4-7 lymphocyte [SD] | 1.16 ± 0.66 | 0.95 ± 0.50 | 0.263 |

^*^These variables were transformed into a log scale
