# Supplementary material for: Temporal changes in the neutrophil to lymphocyte ratio and the neurological progression in cryptogenic stroke with active cancer
Source: PLoS One. 2018 Mar 16;13(3):e0194286. doi: 10.1371/journal.pone.0194286 (PMC5856344; doi:10.1371/journal.pone.0194286)
Supplement: S2 Table — (DOCX) [file pone.0194286.s002.docx]

**S2 Table Multivariate analysis of the possible predictors of END**

|  | **Univariate analysis** | | **Model 1^a^** | | **Model 2^b^** | |
| --- | --- | --- | --- | --- | --- | --- |
|  | **OR** | ***P*** | **aOR** | ***P*** | **aOR** | ***P*** |
| Infection | 1.10 [0.27-4.45] | 0.898 | … | … | 0.32 [0.06-1.87] | 0.208 |
| Initial DWI volume* | 1.58 [1.06-2.36] | 0.026 | 1.25 [0.82-1.91] | 0.291 | 1.25 [0.80-1.98] | 0.329 |
| D 1-3 NLR* | 3.87 [1.65-9.09] | 0.002 | 3.02 [1.17-7.80] | 0.023 | 3.44 [1.26-9.40] | 0.016 |

DWI = Diffusion weighted imaging, NLR = Neutrophil to lymphocyte ratio

^a^Adjusted for initial DWI volume score and D 1-3 NLR

^b^Adjusted for initial DWI volume, D 1-3 NLR, and infection event

^C^This variable was transformed into a log scale
