# Supplementary figures and images for: Temporal changes in the neutrophil to lymphocyte ratio and the neurological progression in cryptogenic stroke with active cancer
Source: PLoS One. 2018 Mar 16;13(3):e0194286. doi: 10.1371/journal.pone.0194286 (PMC5856344; doi:10.1371/journal.pone.0194286)

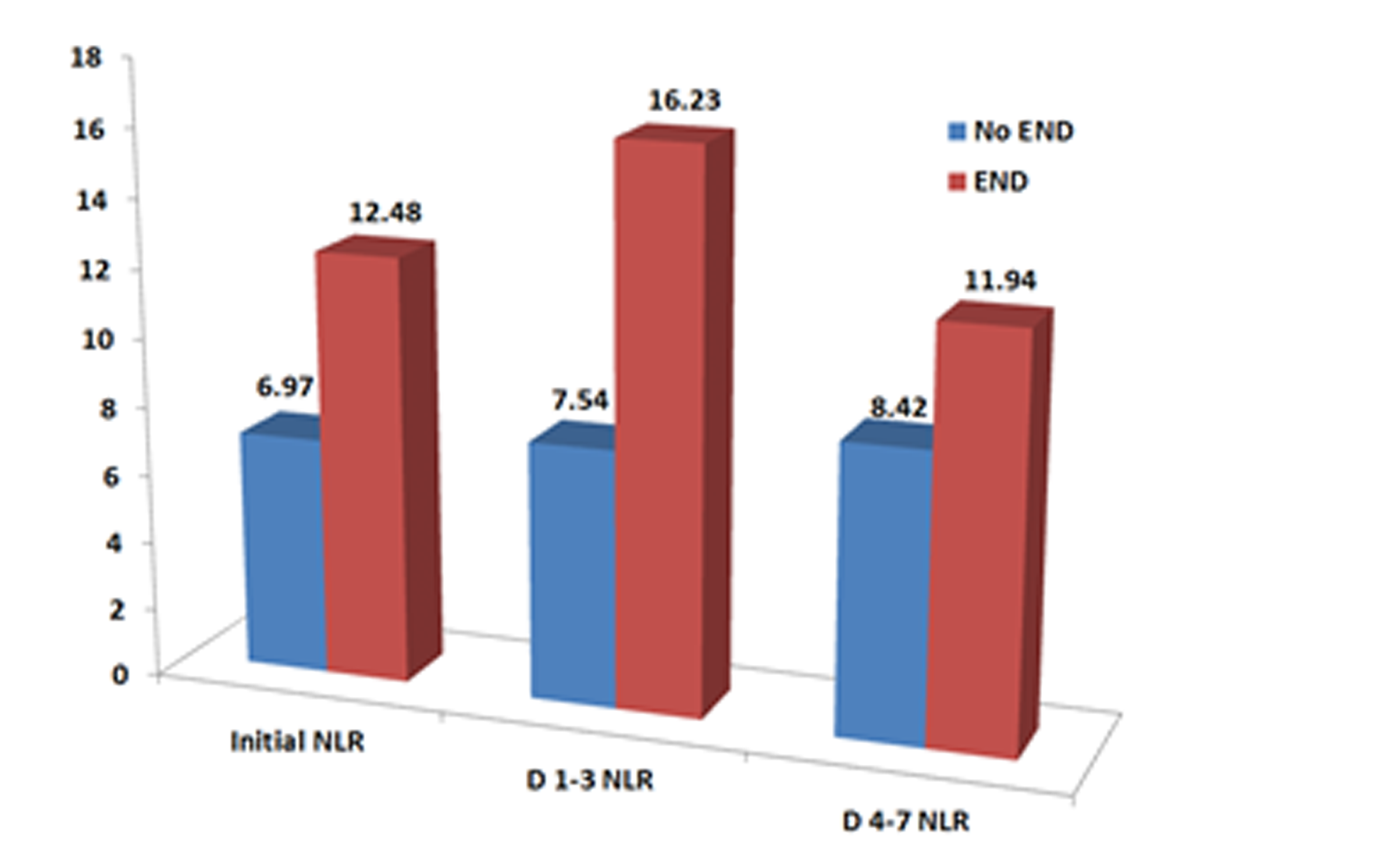

Supplement: S1 Fig — (TIF) [file pone.0194286.s003.tif]

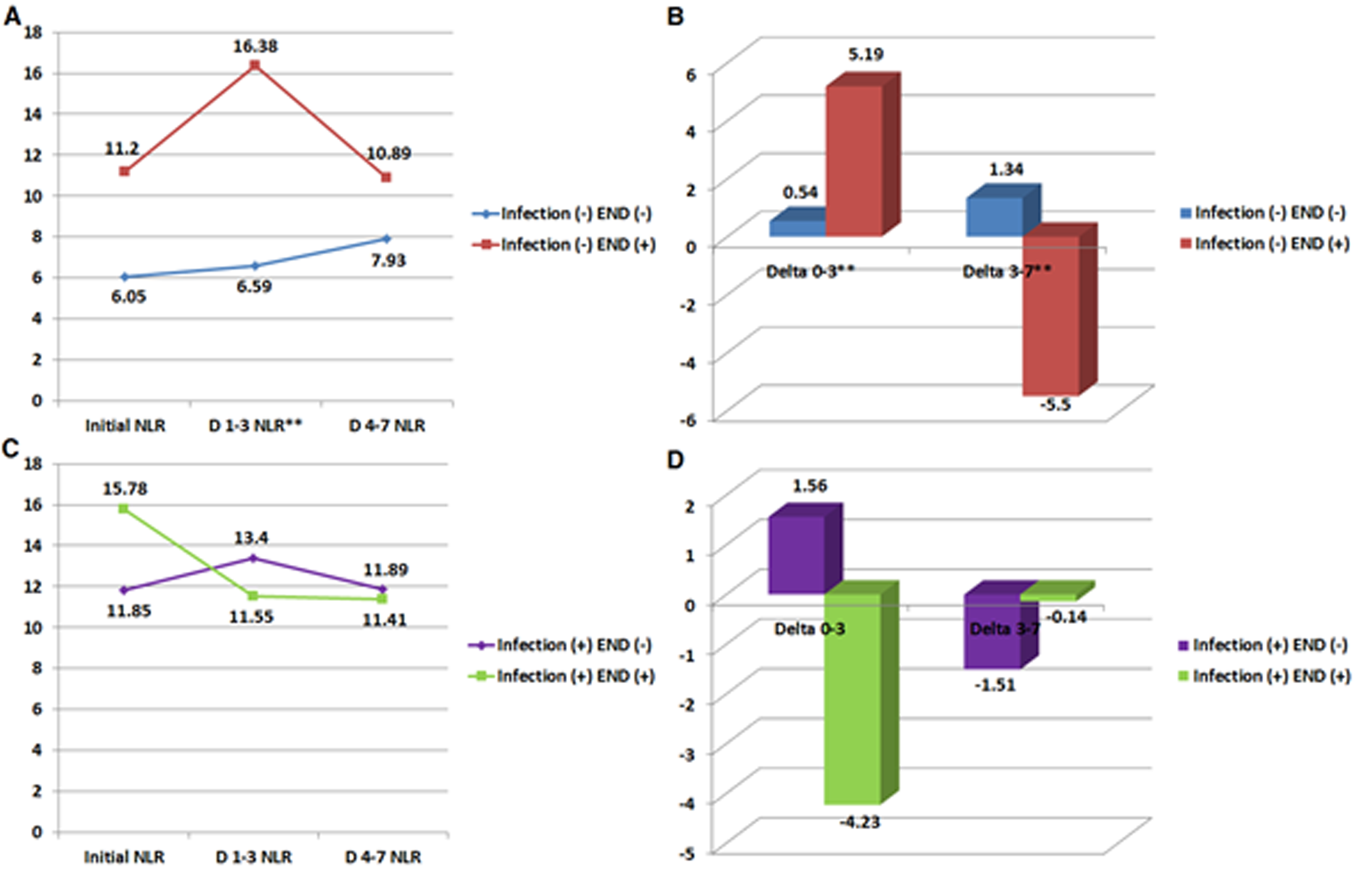

Supplement: S2 Fig — The END group showed a consistently higher NLR than the non-END group, especially the D 1–3 NLR (Mann-Whitney test, P = 0.001), with a tendency toward increase at D 1–3 (A, B). However, these differences were affected by infection events during hospitalization (C, D). (TIF) [file pone.0194286.s004.tif]
